# Cannabigerol Is a Potential Therapeutic Agent in a Novel Combined Therapy for Glioblastoma

**DOI:** 10.3390/cells10020340

**Published:** 2021-02-05

**Authors:** Tamara T. Lah, Metka Novak, Milagros A. Pena Almidon, Oliviero Marinelli, Barbara Žvar Baškovič, Bernarda Majc, Mateja Mlinar, Roman Bošnjak, Barbara Breznik, Roby Zomer, Massimo Nabissi

**Affiliations:** 1 Department of Genetic Toxicology and Cancer Biology, National Institute of Biology, 1000 Ljubljana, Slovenia; metka.novak@nib.si (M.N.); barbara.zvar-baskovic@nib.si (B.Ž.B.); bernarda.majc@nib.si (B.M.); mateja.mlinar@nib.si (M.M.); barbara.breznik@nib.si (B.B.); 2 Faculty of Chemistry and Chemical Technology, University of Ljubljana, 1000 Ljubljana, Slovenia; 3 Jožef Stefan International Postgraduate School, 1000 Ljubljana, Slovenia; 4 School of Pharmacy, Experimental Medicine Section, University of Camerino, 62032 Camerino, Italy; milagros.penaalmidon@studenti.unicam.it (M.A.P.A.); oliviero.marinelli@unicam.it (O.M.); massimo.nabissi@unicam.it (M.N.); 5 Department of Neurosurgery, University Medical Centre Ljubljana, 1000 Ljubljana, Slovenia; roman.bosnjak@kclj.si; 6 MGC Pharmaceuticals d.o.o., 1000 Ljubljana, Slovenia; roby@mgcpharma.com.au

**Keywords:** apoptosis, cannabinoids, cannabigerol, cannabidiol, delta-9-tetrahydrocannabinol, glioblastoma, invasion, temozolomide

## Abstract

**Simple Summary:**

Among primary brain tumours, glioblastoma is the most aggressive. As early relapses are unavoidable despite standard-of-care treatment, the cannabinoids delta-9-tetrahydrocannabinol (THC) and cannabidiol (CBD) alone or in combination have been suggested as a combined treatment strategy for glioblastomas. However, the known psychoactive effects of THC hamper its medical applications in these patients with potential cognitive impairment due to the progression of the disease. Therefore, nontoxic cannabigerol (CBG), being recently shown to exhibit anti-tumour properties in some carcinomas, is assayed here for the first time in glioblastoma with the aim to replace THC. We indeed found CBG to effectively impair the relevant hallmarks of glioblastoma progression, with comparable killing effects to THC and in addition inhibiting the invasion of glioblastoma cells. Moreover, CBG can destroy therapy-resistant glioblastoma stem cells, which are the root of cancer development and extremely resistant to various other treatments of this lethal cancer. CBG should present a new yet unexplored adjuvant treatment strategy of glioblastoma.

**Abstract:**

Glioblastoma is the most aggressive cancer among primary brain tumours. As with other cancers, the incidence of glioblastoma is increasing; despite modern therapies, the overall mean survival of patients post-diagnosis averages around 16 months, a figure that has not changed in many years. Cannabigerol (CBG) has only recently been reported to prevent the progression of certain carcinomas and has not yet been studied in glioblastoma. Here, we have compared the cytotoxic, apoptotic, and anti-invasive effects of the purified natural cannabinoid CBG together with CBD and THC on established differentiated glioblastoma tumour cells and glioblastoma stem cells. CBG and THC reduced the viability of both types of cells to a similar extent, whereas combining CBD with CBG was more efficient than with THC. CBD and CBG, both alone and in combination, induced caspase-dependent cell apoptosis, and there was no additive THC effect. Of note, CBG inhibited glioblastoma invasion in a similar manner to CBD and the chemotherapeutic temozolomide. We have demonstrated that THC has little added value in combined-cannabinoid glioblastoma treatment, suggesting that this psychotropic cannabinoid should be replaced with CBG in future clinical studies of glioblastoma therapy.

## 1. Introduction

Glioblastoma (GB) is one of the most aggressive cancers, and unfortunately, the most frequent among brain tumours. It is characterized by distinct histological features such as necrosis, vascular proliferation, and pleomorphism [1]. Its poor prognosis is due to two major reasons: (1) the diffuse infiltration of highly invasive individual GB cells into the brain parenchyma [2,3,4], which prevents complete tumour resection, and (2) the high resilience of brain tumour-initiating cells, i.e., glioblastoma stem cells (GSCs). These cells most likely evolve from normal neural stem cells or by dedifferentiation (reverse differentiation) of any of their progenitors along with the accumulation of oncogenic mutations during gliomagenesis [5,6,7]. Van Meir et al. [5] suggest that the histological variety of GSC origin drives the development of GB into four different genetic subtypes with distinct driver mutations. Finally, their concomitant appearance in a tumour results in what is termed “intra-tumour heterogeneity”, which is associated with an even worse prognosis of patient survival vs. that of patients with a single GB subtype. The simultaneous targeting of multiple hallmarks of glioma malignancy, such as its rapid growth/proliferation and invasion, and GSC stemness, is challenging. There is a common understanding that this can only be achieved by combined treatment [7,8]. Indeed, the standard-of-care therapy for GBs involves a combination of irradiation and the alkylating agent temozolomide (TMZ), resulting in clinically observed tumour regression [8,9]. This treatment [10] prolongs patient survival by approximately 2 months, and the mechanism of action of TMZ is still not completely understood [9,11].

As combined cancer targeting represents a new wave of cancer treatment [12], cannabis botanicals are being proposed for additional GB treatment. Their inherent polypharmaceutical properties offer distinct advantages over current treatments [13] and could complement standard-of-care treatments. Nevertheless, the positive anticancer effects and minimal side effects of cannabinoids need to be considered. To date, studies on the medical applications of cannabinoids have demonstrated that their off-target effects are not as toxic as those of chemotherapeutics [14,15]. Furthermore, cannabinoids are already widely used for the beneficial palliative treatment of GB patients [16]. The novel use of cannabinoids as anticancer agents is based on a large body of literature, which has demonstrated tumour-specific, cytostatic/cytotoxic effects in experimental models and in humans [17], including GB patients [18].

Cannabinoids are terpenophenolic compounds that are present in the cannabis plant in different concentrations; cannabigerolic acid is the precursor compound of delta-9-tetrahydrocannabinol (THC), cannabidiol (CBD), and CBG (cannabigerol) (Figure S1). However, CBG is present in very low amounts of less than 10% of the cannabinoid fraction in the cannabis plant [19] and has thus been neglected for decades as a medical compound. THC and CBD have been the most investigated in GB patients [14] because both increase the survival of cancer patients. Various cannabinoids have already been used as either (a) natural or synthetic THC alone or together with TMZ in patients with recurrent GBs in clinical trials (CT-NCT01812603, CT-NCT01812616); (b) CBD alone (CT-NCT02255292) or together with TMZ [20]; or (c) an equimolar THC:CBD combination drug (e.g., Sativex or Nabiximol), which is approved in over 30 countries for the management of (cancer-associated) pain. A recent review by Afrin et al. [15] provides more detailed information on several cancer-related phase I/II clinical trials. Recent studies suggest that THC induces cancer cell apoptosis and cytotoxicity by binding predominantly to the cannabinoid CB1 receptor and partially to CB2 [21,22,23,24], which are highly expressed in GBs. Non-THC cannabinoids also modulate the activity of various G-protein-coupled receptors (e.g., GPR55, GPR3, GPR6, and GPR12), transient receptor potential channels of the vanilloid subtype (e.g., TRPV1/2, TRPM8), and the peroxisome proliferator-activated receptor alpha [25,26,27]. While the exact mechanism is more complex and beyond the scope of this report, it is known that THC, CBD, and CBG signalling pathways interfere with oncogenic cellular signalling. However, it is still unclear how the cannabinoid-responsive signalling pathways interact among themselves, due to the fact that they can interact with several of the above-mentioned receptors simultaneously and by different modes of interaction.

Although CBG was first described by Baek et al. [28] in 1996, it has not attracted much attention for targeting cancer. Several pathological disorders and the potential medical use of CBG have recently been reviewed by Deiana [29], including very limited yet promising research on its anticancer activity [30,31]. CBG was reported to reduce cell proliferation in several cancer cell lines, including human breast [31,32], prostate, and colorectal carcinoma, gastric adenocarcinoma, C6-rat glioma, rat basophilic leukaemia, and transformed thyroid cells, as reviewed by Ligresti et al. [33]. Interestingly, prostate carcinoma cells only responded to CBD and CBG, whereas THC did not elicit any antiproliferative effects [25,34,35]. CBG readily crosses the blood-brain barrier [36], and plasma and brain pharmacokinetics of CBG have demonstrated great similarities in rats and mice. So far, there are no reports on its effects in GBs.

In the present study, we aimed to elucidate the potential of the least investigated cannabinoid, CBG, to target several hallmarks of glioma progression, such as fast growth/proliferation, invasiveness, GSCs, and resistance to apoptosis. We have compared the effects of CBG with those of THC and CBD and report on (i) reduced GB and GSC viability (cytotoxicity), (ii) apoptosis induced by CBG vs. THC and CBD in selected GB and GSC lines, (iii) the inhibitory effect of CBG on the extent of invasion in GB spheroids, and (iv) assessments of the optimal combination of non-THC cannabinoids with THC and TMZ regarding GB cell and GSC cytotoxicity and the inhibition of GB cell invasiveness.

## 2. Materials and Methods

### 2.1. Cannabinoids

The following purified extracts from the cannabis plant were provided by MGC Pharmaceuticals Ltd. (Ljubljana, Slovenia): Δ^9^-tetrahydrocannabinol (THC), cannabidiol (CBD), and cannabigerol (CBG). The purity of the compounds was controlled by high-performance liquid chromatography (Table S1). Consecutive batches were of a similar degree of purity, and the concentration ranges in organic solvents (dimethyl sulfoxide (DMSO) for CBD and THC, and ethanol for CBG) were as follows: THC (15674–17294 mg/mL), CBD (14367–14895 mg/mL), and CBG (5772–7369 mg/mL). The stability of the active compounds was tested regularly. For GB cell toxicity tests, THC and CBD were diluted in 100% DMSO to a stock concentration of 50 mM, whereas CBG was diluted in ethanol to a stock concentration of 20 mM.

### 2.2. Cell Cultures

The established differentiated human GB cell lines U87 and U373 were purchased from the American Type Culture Collection (USA), and the T98 cell line was obtained from the European Collection of Cell Cultures (ECACC, Salisbury, UK). All of the cell lines were grown in high-glucose Dulbecco’s modified Eagle medium (DMEM; GE Healthcare, IL, USA), supplemented with 10% (*v*/*v*) heat-inactivated foetal bovine serum (FBS), 2 mM L-glutamine, 100 IU/mL penicillin, and 100 µg streptomycin, as described in Kološa [37] and Breznik [38]. The GSCs NCH644 and NCH421k were first obtained as a gift from Prof. Christel Herold-Mende (University of Heidelberg, Heidelberg, Germany) and by Cell Lines Service (GmbH, Berlin, Germany). These were grown as spheroid suspensions in complete neurobasal medium (Invitrogen, Life Technologies, Carlsbad, CA, USA), 20 ng/mL bFGF (beta fibroblast growth factor), and EGF (epidermal growth factor) (both from Invitrogen, Life Technologies, Carlsbad, CA, USA). All of the cell lines were maintained at 37 °C with 5% CO_2_ and 95% humidity and were tested for mycoplasma contamination using a MycoAlert Mycoplasma Detection Kit (Lonza Pharma & Biotech Ltd., Bend, OR, USA).

### 2.3. Establishment of Primary GB Cells

Glioblastoma surgical biopsies were obtained from consecutively operated patients at the Department of Neurosurgery, University Medical Centre of Ljubljana, Slovenia. The study was approved by the National Medical Ethics Committee of the Republic of Slovenia (approval No. 0120-179 190/2018/4).

Before any treatment (except with dexametason to reduce inflammatory swelling), fresh tumor tissues were stored in the NIB-maintained Gliobank that contains mainly tissues of primary glioblastoma, IDH wild-type, WHO grade 4 and a few astrocytomas, previously called glioblastoma, IDH-mutant, and WHO grade 4 according to EANO classification (European Association of Neuro-Oncology) [39]. Gliobank consists of frozen tumor tissues, primary cultures of GB and GSC glioblastoma (stem) cells, and organoids, all isolated and established from tumor biopsies of glioblastoma patients, the related clinical patient’s data, as well as histological, histochemical, and molecular data. For the establishment of mature and differentiated GB cell lines, fresh GB tumour tissue samples were minced (by scalpels) in high-glucose DMEM supplemented with 10% FBS, 2 mM L-glutamine, and penicillin-streptomycin, and then seeded onto six-well plates. After reaching confluency, the cells were detached with a 0.25% trypsin-EDTA solution in phosphate-buffered saline (PBS) and transferred to T25 cell culture flasks. Cells passaged 2–3 times in this manner were transferred to T75 culture flasks and expanded for subsequent analyses.

### 2.4. Establishment of GSC Lines

Freshly resected human GB tumours were prepared as previously described [39]. The tumour mass was minced into small pieces, trypsinized into a single-cell suspension using 0.25% trypsin-EDTA solution (Sigma Aldrich, St. Louis, MO, USA), and collected by low-speed centrifugation (1000 rpm for 60 s). The cell solution was further filtered through a nylon mesh with 40 μm pores (Falcon cell strainer, nylon; Becton Dickinson, Franklin Lakes, NJ, USA). Single cells were collected and resuspended in stem cell media: neurobasal medium containing 2 mM L-glutamine, 1 × penicillin/streptomycin, 1 × B-27, 1 U/mL heparin (Sigma-Aldrich, St. Louis, MO, USA), and 20 ng/mL bFGF and EGF (both from Invitrogen, Life Technologies, Carlsbad, CA, USA). Cells were cultured on agar-coated T25 flasks until spheres with diameters of 200 μm were formed. GSCs were frozen in stem cell media with 10% DMSO for further analyses. The GSC line was authenticated by immunolabelling of several stem cell markers, as described by Podergajs [40] using immunofluorescence and flow cytometry.

### 2.5. Cell Viability Assay

Cell viability was determined with MTT assay, i.e., 3-(4,5-dimethylthiazol-2-yl)-2,5-diphenyltetrazolium-bromide (Sigma-Aldrich, St. Louis, MO, USA), for GB differentiated cells and with MTS 3-(4,5-dimethylthiazol-2-yl)-5-(3-carboxymethoxyphenyl)-2-(4-sulfophenyl)-2H-tetrazolium salt (Promega, Madison, WI, USA) for GSCs. Differentiated GB cells and GSCs were seeded onto 96-well plates at a density of 5000 and 8000 to 10000 cells/well, respectively. Cells were treated with different concentrations (0.16–50 µM) of the cannabinoids CBG, CBD, and THC alone or combined with TMZ (Sigma-Aldrich, St. Louis, MO, USA) at final concentrations of 100 µM or 400 µM. Samples contained the same amount of DMSO (≤ 0.4%, *v*/*v*) for CBD and THC and ethanol (0.24%, *v*/*v*) for CBG under all of the treatment conditions. Cell viability was measured after 48 h of incubation by adding MTT or MTS reagent when spheroids were tested. All dose-response experiments were performed in triplicate, i.e., with three biological repeats. Absorbance was measured as the change in optical density (ΔOD 570/690 nm) using a microplate reader (Synergy™ HT, Bio-Tec Instruments Inc., Winooski, VT, USA). Cell viability and IC50 µM values were calculated using dose response curves, plotted using GraphPad Prism software. The factor of inhibitory concentration (FIC), which discriminates between the additive and synergistic effects of two drugs, was calculated using method number 2 from Deng et al. [41], who relied on Orhan [42], which is based on the concentrations at which the cannabinoids (CBG and CBD) produce half-maximal inhibition (IC50 µM values). Thus, the CBD concentration was fixed, and the dose-response curve of the inhibitory effects of CBG, added at serial (half log_1_) dilutions, was plotted. The IC50 of CBG (in the presence of the fixed CBD concentration) was then calculated using GraphPad Prism software. The FIC efficacy of the combination was calculated, and the following was defined: synergy (FIC < 0.5), additivity (0.5 < FIC < 4), and antagonism (FIC > 4). Statistical analyses were performed using GraphPad Prism software, using one-way ANOVA with Bonferroni’s post-hoc test.

### 2.6. Three-Dimensional (3D) Tumour Spheroid Invasion Assay

Cells were labelled before 3D tumour spheroid invasion assay by Vybrant® Cell-Labeling solution (Thermo Fisher Scientific, Inc., Waltham, MA, USA) with the following spectral maxima: DiO excitation at 484 nm, and emission at 501 nm. The 1 mM stock solution was stored at −20 °C. The U373 and T98 cells were removed from the culture dish using 0.1% trypsin (Sigma-Aldrich, St. Louis, MO, USA), and then counted. Next, 0.5 × 10^6^ cells were incubated for 10 min at 37 °C with a 5 µM dye solution in 1 mL of serum-free culture medium. After incubation, the cells were washed twice in PBS and resuspended in 1 mL of cell medium with serum. U87 dsRED cells and NCH421kGFP were labelled as described by Breznik et al. [38]. The GB cell lines T98, U87, and U373 were then seeded onto 96-well plates (5 × 10^3^ cells/well; Corning, NY, USA) in high-glucose DMEM containing 4% methylcellulose, and NCH421k cells were seeded onto 96-well plates (5 × 10^3^ cells/well; Corning, NY, USA) in complete Neurobasal Medium (Invitrogen, Life Technologies) containing 2 mM L-glutamine, 1 × penicillin/streptomycin, 1 × B-27 (Invitrogen, Life Technologies), 1 U/mL heparin (Sigma-Aldrich, St. Louis, MO, USA), 20 ng/mL bFGF and EGF (both from Invitrogen, Life Technologies), and 4% methylcellulose. The cells were centrifuged at 850× *g* for 60 min and incubated at 37 °C and 5% CO_2_ for four days (U87), two days (U373 and T98), and three days (NCH421k) to form one spheroid in each well. These spheroids were treated with CBG (10, 25, and 50 µM), CBD (2, 5, and 10 µM), and TMZ (100, 200, and 400 µM). The spheroids were then covered with 5 mg/mL Matrigel matrix (Corning, NY, USA). The invasion distance was measured after seven days for U87 cells and five days for U373 and T98 cells. We measured the extent of invasion with the fluorescence microscope NIKON-Eclipse Ti at 4× magnification. The invasion area, normalized to spheroid diameter, was determined by ImageJ software as described in Breznik et al. [38] and Hiram et al. [43].

### 2.7. Immunofluorescence of GSC Spheroids

The 3D GSC spheroids were washed with PBS, fixed in ice-cold methanol (Sigma-Aldrich, St. Louis, MO, USA) for 15 min at room temperature (T), and incubated for 15 min in 0.1% Triton X-100/1% FBS/PBS at room temperature 22 °C, for membrane permeabilization. The spheroids were stained for 30 min at room temperature with the following antibodies: CB1 (ab23703, Abcam, Cambridge, UK, dilution 1:200) and CB2 (ab189841, Abcam, Cambridge, UK, dilution 1:500). Negative control staining was performed with the blocking peptides CB1 (ab50542, Abcam, Cambridge, UK, dilution 1:80) and CB2 (ab45941, Abcam, Cambridge, UK, dilution 1:50), which bind specifically to the target antibody epitope at a 10-fold higher concentration than the primary antibodies. Spheroids were stained with an Alexa Fluor 488^®^-conjugated secondary antibody (1:200; Invitrogen, Life Technologies, Carlsbad, CA, USA) for 30 min at room T. For nuclear staining, the spheroids were incubated with Hoechst 33342 dye (1:1000, Invitrogen, Life Technologies, Carlsbad, CA, USA) for 5 min at room T. The spheroids were then mounted with AntiFade reagent (Invitrogen, Life Technologies, Carlsbad, CA, USA) and analysed with a confocal microscope (Leica DFC 7000 T, Wetzlar, Germany).

### 2.8. Immunocytochemistry

Immunohistochemistry was performed using antibodies against CB1 (ab23703, Abcam, Cambridge, UK, dilution 1:200), CB1 peptide (ab50542, Abcam, Cambridge, UK, dilution 1:80), CB2 (ab189841, Abcam, Cambridge, UK, dilution 1:500), and CB2 peptide (ab45941, Abcam, Cambridge, UK, dilution 1:50). Before incubation with antibodies, non-specific binding sites were blocked with 1% bovine serum albumin with 2% goat serum in PBS overnight at 5–7 °C. The sections were incubated with biotinylated secondary antibody followed by horseradish peroxidase-conjugated streptavidin (Cell Signaling Technology, Danvers, MA, USA). The sections were further incubated with 2,4-diaminobenzidine substrate and counterstained with haematoxylin. To achieve high antibody specificity, we used CB1 and CB2 blocking peptides that bind specifically to the target antibody epitope at a 10-fold higher concentration than the primary antibodies.

### 2.9. Cell Cycle Analyses

Cells (3 × 10^4^ cells/mL) were incubated with the cannabinoids or vehicle (solvent) for 48 h. Cells were fixed for 1 h by adding ice-cold 70% ethanol, after which they were washed with buffer (PBS, 2% FBS, and 0.01% NaN_3_). This was followed by incubation with 100 μg/mL ribonuclease A solution (Sigma-Aldrich, St. Louis, MO, USA) for 30 min at 37 °C and staining for 30 min at room T with propidium iodide (20 μg/mL; Sigma-Aldrich, St. Louis, MO, USA). The cells were centrifuged, the supernatant was removed, and cell pellets were re-suspended in 500 mL of PBS. Flow cytometry analyses were performed by using linear amplification.

### 2.10. Apoptosis Analyses

The assessment of cannabinoid-induced apoptosis was performed by flow cytometry. Cells (100,000 cells for each treatment) were treated with IC50 concentrations of CBG, CBD, and THC, harvested after 48 h, pelleted, and washed with 1 × PBS. Early/late apoptotic cells were detected by staining with Annexin-V-FITC (Miltenyi Biotech, Bergisch Gladbach, Germany) for 15 min in the dark at room T and with 1 µg/mL of propidium iodide solution (100 μg/mL; Miltenyi Biotech, Bergisch Gladbach, Germany). Stained cells were analysed using a flow cytometer (BD FACSCalibur, BD Biosciences, Allschwil, Switzerland) and analysed by the CellQuest software (BD Biosciences, Allschwil, Switzerland). Vehicles/solvents, i.e., diluted DMSO and ethanol (0.25% *v*/*v*), served as negative controls, whereas the well-known apoptotic agent staurosporine (Sigma-Aldrich, St. Louis, MO, USA) (10 μM, 4 h) served as a positive control for apoptosis, as described by Kenig et al. [44].

### 2.11. Caspase-3-Dependent Apoptosis

CBG-induced apoptosis was measured by western blot of caspase-3 activation (proteolytic cleavage) assay. Lysis buffer (1 M TRIS pH 7.4, 1 M NaCl, 10 mM EGTA, 100 mM NaF, 2% deoxycholate, 100 mM EDTA, 10% TritonX-100, glycerol, 10% SDS, 1 M Na_2_P_2_O_7_, 100 mM Na_3_VO_4_, 100 mM PMSF (phenylmethylsulfonyl fluoride), a commercial cocktail of protease inhibitors, and H_2_O) was used, and 40 µg of protein was obtained and then separated on 12% SDS polyacrylamide gels in electrophoresis chambers (BioRad Labs, Feldkirchen, Germany). The proteins were transferred onto Hybond-C extra membranes (GE Healthcare), blocked with 5% low-fat dry milk in 0.1% Tween 20 in PBS for 1 h, immunoblotted with rabbit anti-caspase-3 (1:1000, Cell Signaling) and mouse anti-glyceraldehyde-3-phosphate dehydrogenase (GAPDH, 1:3000, OriGene, Rockville, MD, USA) antibodies overnight, and then incubated with their respective HRP (horseradish peroxidase)-conjugated anti-rabbit and anti-mouse (1:2000, Cell Signaling) antibodies for 1 h. Peroxidase activity was visualized with a LiteAblot^®^PLUS or TURBO (EuroClone, Milan, Italy) kit, and densitometric analysis was performed with Chemidoc using the Quantity One software (Bio-Rad, Feldkirchen, Germany).

## 3. Results

### 3.1. The Cannabinoids CBG, CBD, and THC Affect the Viability of Primary GB Cells and GSCs

**Figure 1:**
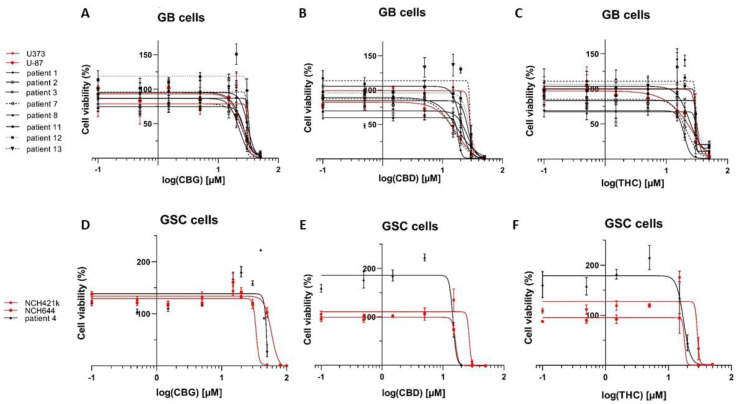
Cannabinoids reduce viability of GB and GSC cells. CBG (**A**,**D**), CBD (**B**,**E**), and THC (**C**,**F**) impaired the viability of differentiated GB cells and GSCs. Dose responses of cell viability measured by MTT assay (y-axis) and different CBG, CBD and THC concentrations increasing in the range of 0.1–50 µM (x-axis log scale) on established GB cells (U87 and U373) (**A**–**C**; red lines) in comparison to primary patient-derived GB cells (**A**–**C**; black lines). Established GSC lines NCH421k and NCH644 (**D**–**F**; red lines) are compared to primary patient-derived GSCs (**D**–**F**; black line) after 48 h of a single treatment with cannabinoids. Data are expressed as mean ± SE (*n* = 3–5 independent biological experiments, each in technical triplicate). Vehicles comprised ≤ 0.1% (*v*/*v*) DMSO for THC and CBD and 0.24% (*v*/*v*) ethanol for CBG. The methodology taken from Deng et al. [41] is described in great detail in Material and Methods (Section 2.5).

We investigated the effects of CBG, CBD, and THC on the viability of GSCs and terminally differentiated GB cell lines, using the inhibition of mitochondrial dehydrogenases activity MTT and MTS assays for spheroid cultures, after 48 h of treatment. The viability of both GB cell lines and GSCs were significantly reduced by all three cannabinoids (Figure 1). In three of the established and ten of the primary patient-derived GB cell lines, CBG reduced GB cell viability in a concentration range of 22–32 μM (IC50 28.1 ± 1.1 μM), which was similar to THC (IC50 27.9 ± 1.8 μM). Tested on the same cell lines, CBD was significantly more cytotoxic with IC50 22.0 ± 2.1 μM, (*p* < 0.0), compared to CBG and THC, that were not statistically different among various GB lines. In GSCs, the IC50 values were 59 ± 15 μM (CBG), 20 ± 4 μM (CBD), and 23 ± 3 μM (THC) and were of the same order of magnitude as in the GB lines. Overall, CBD exerted the strongest inhibitory effect on the viability of both GB cells and GSCs (Table 1). Our quantitative results confirm previous results on the effects of CBD and THC on GB cell viability [24] and demonstrate a novel effect on GSC viability as well. Furthermore, our results demonstrate that CBG reduces the viability of GB and GSC lines with a similar potency as CBD and THC.

### 3.2. CBG Exerts a Cytostatic Effect

**Figure 2:**
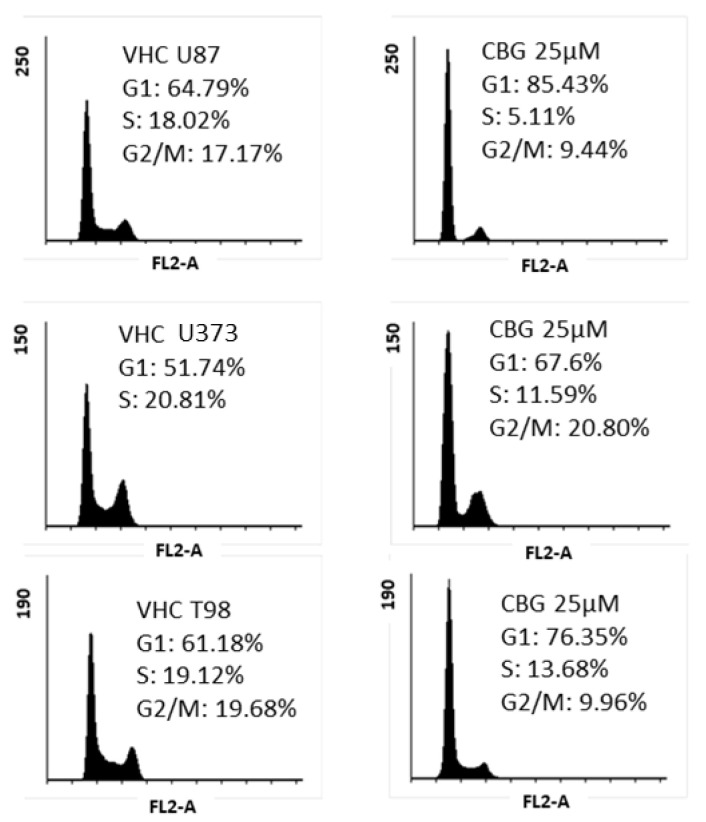
CBG-induced cell-cycle arrest in the G1 phase in GB cell lines. The cell lines U87, U373, and T98 were treated with 25 μM CBG twice within 48 h. The representative cell cycle distribution was analysed by flow cytometry, as described in the Materials and Methods sections (Section 2.9). The upper panel represents the shift in cell-cycle phase distribution (y axis: percentage of cells) vs. vehicle (left panels, comprising 0.24% (*v*/*v*) ethanol) after CBG treatments of U87 cells (upper panel), U373 cells (middle panel), and T98 cells (lower panel). Accumulation of cells in the G1 phase, reducing the percentage of cells in S phase and G2/M phase, was observed in all three established GB cell lines.

As CBG exhibited cytotoxicity, we further investigated whether CBG exerts cytostatic effects on three different established GB cell lines U87, U373, and T98. To enhance the effects of CBG, cells were treated with 25 µM of CBG within 48 h. CBG induced an accumulation of cells in the G1 phase with a larger effect in U87, where the G1 phase increased for 20.6 % (by 7.6%), followed U373 cells with G1 phase increased for 15.7% (by 7.6%) and in T98 increased for 14.5% (by 6.7%). Reducing the % of cells in the S1 and G2/M phase was seen in all three cell lines (Figure 2).

### 3.3. The Cannabinoids CBG, CBD, and THC Affect Apoptosis of Primary GB Cells and GSCs

**Figure 3:**
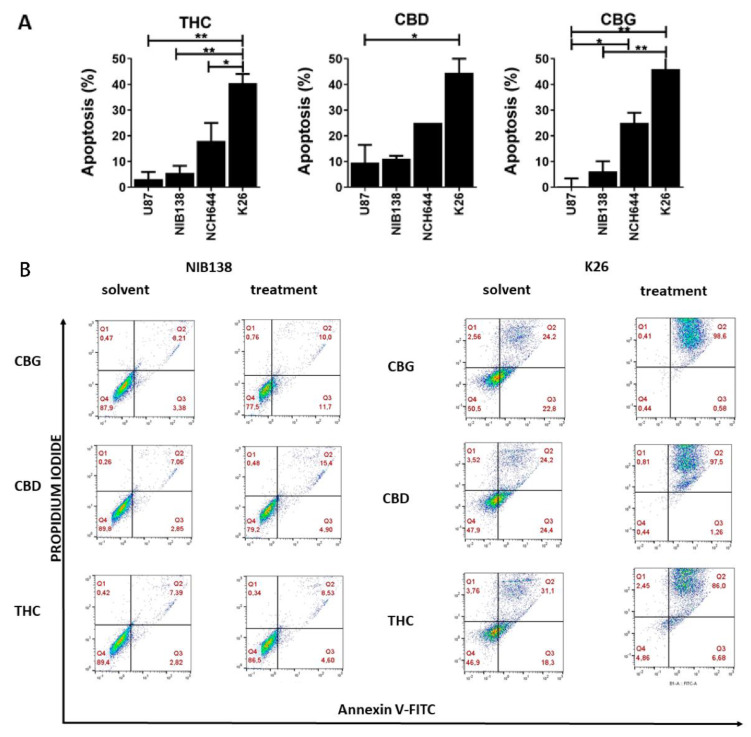
Apoptosis determination of GB and GSC cells by flow cytometry. The cells were treated with IC50 concentrations of CBG, CBD, and THC for 48 h. (**A**) The summary graphs represent the percentage of cells in early and late apoptosis after cannabinoid treatment (mean ± S.E.M.). (**B**) Cells were labelled with Annexin V-FITC (x-axis) and propidium iodide (PI) (y-axis) and analysed by flow cytometry as described in more detail in Material and Methods (Section 2.10). The cell distributions based on cytometry analysis are presented for the GB cells NIB138 (left) and GSC line K26 (right) after CBG, CBD, and THC treatment. Additionally, such data for the established GB cells U87 and NCH644 are presented in Figure S2. Dot blots represent the results from one representative biological repeat. Error bars represent mean ± S.E.M. Statistical analyses were performed using GraphPad Prism software, using one-way ANOVA (* *p* < 0.05, ** *p* < 0.01).

We further tested whether CBG induces cell death via late apoptosis in the following established differentiated GB line (U87) and GSC line (NCH644) as well as patient-derived GB cells (NIB138) and GSCs (K26). These representative lines were treated with CBG, CBD, and THC at the corresponding IC50 concentrations (μM), based on the cell viability assay (Table 1). To validate the experimental conditions, staurosporin, standard pro-apoptotic coumpound treatment (10 μM, 4 h) served as a positive control [44]; the data is not shown. Most of the cells were negative for propidium iodide, indicating that they were in an early stage of apoptosis, whereas double-positive staining revealed that cells were in a late stage of apoptosis (or necrosis) (Figure 3). CBG induced apoptosis in up to 10 to 18% of differentiated GB cells (Figure 3A), and no or lower levels by CBG and THC. CBD exerted a similar effect on GB and GSC apoptosis as CBG (Figure 3A). Conversely, CBG, CBD, and THC induced GB and GSC apoptosis, which was more prominent in GSCs, as patient-derived GSC K26 cells exhibited 40 to 50% of cell apoptosis (Figure 3B).

Taken together, GSCs are more sensitive than differentiated GB cells against cell death induction by all three cannabinoids. Late apoptosis was induced at concentrations at which half of the cells were viable, as measured by MTT/MTS assays. Interestingly, even in more viable K26 cells line, apoptosis seems to be the major reason for the loss of its viability (at IC50 concentration). This suggests that the observed effect on viability was to a large extent due to GSC apoptosis, whereas the negative effect of cannabinoids on the viability of differentiated non-stem GB cells was also due to cell cycle arrest and possible senescence and other processes reducing mitochondrial activity (such as autophagy).

**Figure 4:**
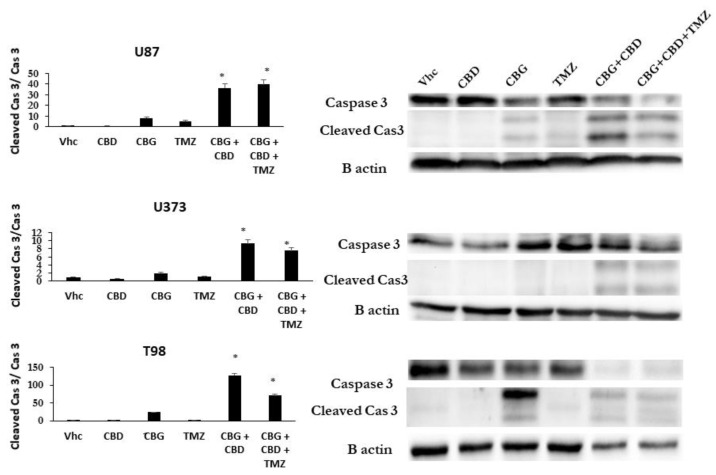
CBG apoptotic pathways processed via induced caspase-3 cleavage in GB cell lines. Apoptosis pathways were analysed by western blot analysis and densitometric quantification of caspase-3 protein levels, as described in Material and Methods (Section 2.11). Representative blots show protein levels of caspase-3 and its cleaved products. Densitometric values were normalized to B actin, which was used as a loading control. Cleaved caspase-3 values were then normalized to non-cleaved caspase-3 and shown on the left graphs as averaged values of three separate experiments (* *p* < 0.05 treated vs untreated cells).

Based on the very similar efficacy of THC and CBG with respect to viability inhibition and apoptosis induction, we focused further experiments on CBD and CBG only. We investigated whether the CBG-induced apoptotic pathway in GB cells is caspase-3-dependent, similar as described for CBD [22]. The GB cell lines U87, U373, and T98 were treated with IC50 concentrations of CBD, CBG, and TMZ alone or in the combinations CBD:CBG, and CBD:CBG:TMZ for 48 h. The apoptotic pathway-induced activation of caspase-3 was quantified by western blot analysis, based on the comparison of cleaved vs. intact caspase 3 immunoreactivity (upper lane, Figure 4). Similar to TMZ treatment alone, CBD alone did not activate caspase-3, in contrast to CBG treatment alone in T98 and U87 cells, but was non-significant in U373 cells under these experimental conditions (Figure 4). However, combined CBD:CBG induced the strongest activation of caspase-3 in all three of the GB cell lines, most prominently in T98 cells (Figure 4). The addition of TMZ to CBD:CBG further increased caspase-3 activation and lysis in the U87 cells, whereas It was slightly and strongly decreased in the U373 and T98 cell lines, respectively, compared to U87 under the same treatment conditions.

### 3.4. Combinations of CBG and CBD Reduced the Viability of GB Cells and GSCs in an Additive Manner

**Figure 5:**
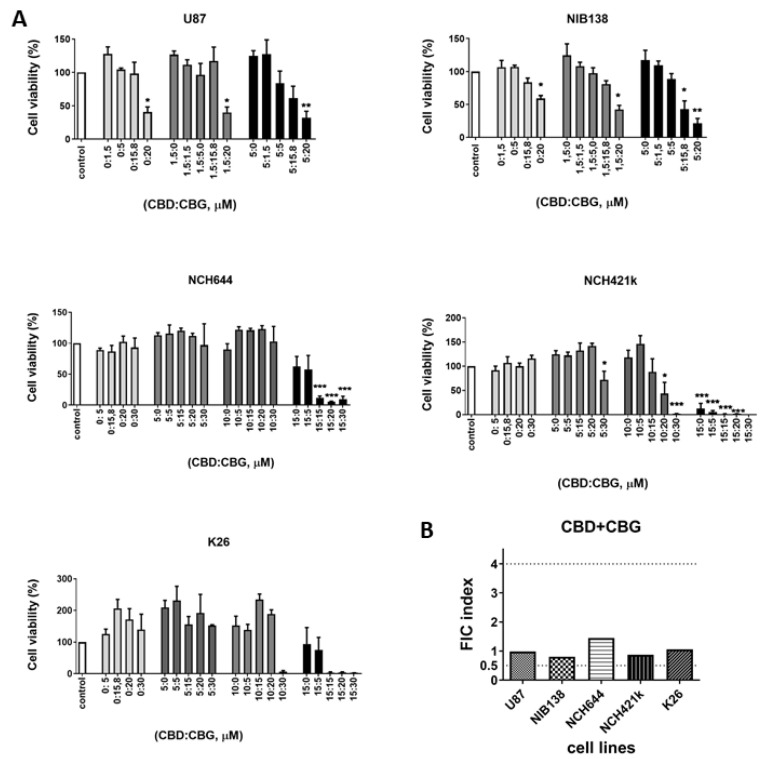
The effects of combined CBD and CBG on the viability of GB cells and GSCs. (**A**) The efficacy of combined CBD and CBG treatment was tested on the following cell lines: U87, patient-derived NIB138, NCH644, NCH421k, and K26. The viability was determined by MTT/MTS assays after 48 h of single treatment as described in Materials and Methods (Section 2.5). White bars represent the untreated controls, and the grey and black bars represent treatments with fixed concentrations of CBD (1.5 and 5 μM for GB lines and 5, 10, and 15 μM for GSC lines); increasing concentrations of CBG (0–20 μM) were added. Error bars represent mean ± S.E.M. Statistical analyses were performed using GraphPad Prism software, using one-way ANOVA (* *p* < 0.05, ** *p* < 0.01, *** *p* < 0.001). Three biological and three technical repeats were performed. Vehicle comprised ≤ 0.4% (*v*/*v*) DMSO for CBD and 0.24% (*v*/*v*) ethanol for CBG. (**B**) The combination responses were determined as the factor of inhibitory concentration (FIC) values between CBD and CBG on GB cells and GSCs. FIC values were calculated from combined CBD:CBG treatment that produced half-maximal effects on the viability of GB cells and GSCs when CBD was fixed at 1.5 and 5 μM for the U87, and NIB138 lines and at 5, 10, and 15 μM for GSCs. Synergy was defined as FIC < 0.5, additivity was defined as 0.5 < FIC < 4, and antagonism was defined as FIC > 4.

The combined effects were studied among CBG, CBD, THC, and TMZ in the following cells: the GB cell line U87, the patient-derived primary GB cell line NIB138, the established GSC lines NCH644 and NCH421k, and the patient-derived GSCs K26. Based on the CBG and CBD IC50 (μM) values, we set up different combinations of the two cannabinoids. CBD was used at 1.5 and 5 μM for GB lines and at 5, 10, and 15 μM for GSCs; the dose-response CBG concentrations were plotted (Figure 5A). For differentiated GB cell lines, a significant response on cell viability was observed with the 5 μM CBD:20 μM CBG, with a molar ratio of 1:4. For GSCs, the most significant effect was observed with the 15 μM CBD:5 μM CBG, with a molar ratio of 3:1 (Figure 5A). The factor of inhibitory concentration (FIC) for the interactive responses between CBD and CBG was calculated, as extensively described in Materials and Methods, Section 2.5, and is based on the combinations that produce half-maximal inhibition (IC50, concentration), according to Deng et al. [41]. FIC analysis revealed that CBD:CBG combinations resulted in an additive response in both GB cells and GSCs (Figure 5B). Noteworthy at very low concentrations, below 10 μM for CBD and 5 μM for CBG increased GSC viability. It is possible that cannabinoids first induce GSC dedifferentiation, as reported by Aguado et al. [45], leading to increased metabolic activity.

### 3.5. Inhibition of GB Cells and GSCs Viability by Combinations of CBG and CBD with THC and TMZ

**Figure 6:**
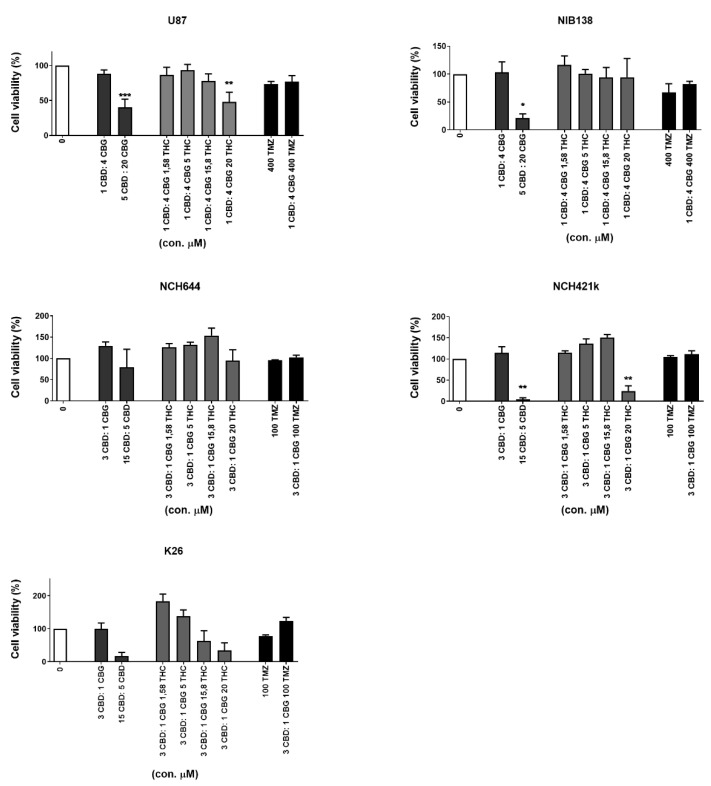
The effects of combined CBD, CBG, THC, and TMZ on the viability of GB cells and GSCs. The GB cell line U87, patient-derived cell lines NIB138 and NCH421k, and GSC lines NCH644 and K26 were exposed to optimized CBD:CBG combinations (see Figure 5) to which increasing THC and two selected TMZ concentrations were added, based on the results of Thorres et al. [24], and treated for 48 h. Light grey bars represent combinations of CBG and CBD with THC, black bars represent TMZ treatment alone or in combination with CBD:CBG, and white bars represent vehicle-treated controls to which all the data are normalized. Error bars represent mean ± S.E.M. Statistical analyses were performed using GraphPad Prism software, using one-way ANOVA (* *p* < 0.05, ** *p* < 0.01, and *** *p* < 0.001). Three biological and three technical repeats were performed. Vehicle comprised ≤ 0.4% (*v*/*v*) DMSO for CBD and 0.24% (*v*/*v*) ethanol for CBG.

To further assess the inhibition of cell viability, we combined CBD and CBG at different ratios, according to Deng et al. [41]. We determined the most efficient molar ratios for CBD:CBG as 1:4 for GB cells and 3:1 for GSCs. CBD:CBG at 5 μM:20 μM efficiently (>50%) and significantly (*p* < 0.05%) inhibited U87 and NIB138 GB cells. To this combinations, THC was added in increasing concentrations, in the range from 1 to 20 μM (Figure 6). At the subtoxic CBD:CBG ratio of 1:4, THC had no effect on CBD:CBG cytotoxicity. However, better efficacy was achieved by increasing the concentration of the CBD:CBG up to 5 μM:20 μM. We conclude that for an effective combined treatment of GB cells addition of THC is not required, as titrating GB cells with the CBD:CBG ratio of 1:4 sufficiently inhibited cell viability.

Similar results were demonstrated for the GSC lines NCH644 and NCH421k at the CBD:CBG ratio of 3:1, which most efficient inhibited cell viability. The addition of THC to subtoxic CBD:CBG concentrations had no efficacy. At or above 15 μM:5 μM of CBD:CBG ratio, GSC cell viability was sufficiently inhibited in all three GSC lines (see the third column from the left in each graph, Figure 6), most prominently in the NCH421k cell line, where THC addition would not be needed to reach 95% viability inhibition. The cytotoxicity of the CBD:CBG combination was also more effective than 100 to 400 μM TMZ alone (Figure 6). We conclude that THC and TMZ do not exert any additive effect on optimized CBD:CBG mixtures, which sufficiently inhibit GSC and GB cell viability at optimised ratios and concentrations.

### 3.6. The Effect of Cannabinoids CBG, CBD and TMZ on Invasion of GB and GSC Cells

**Figure 7:**
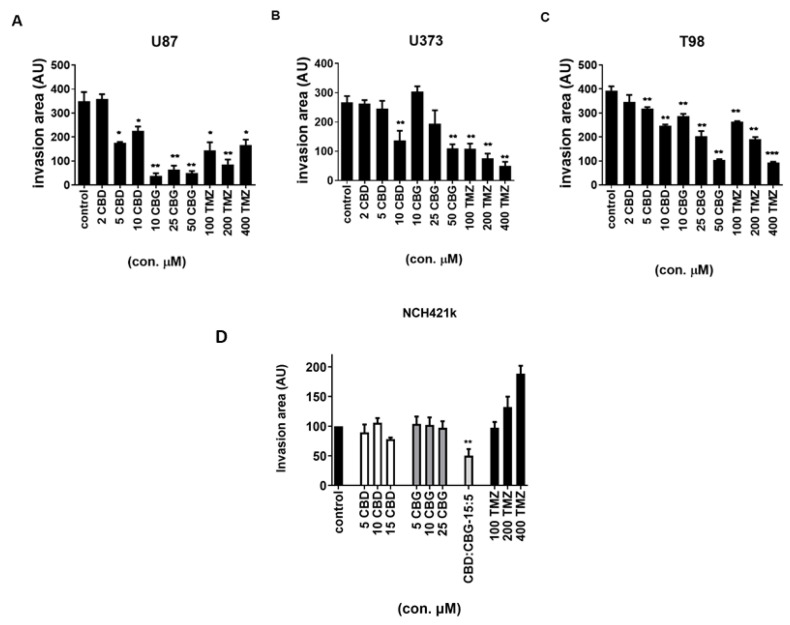
The effect of CBD and CBG on GB and GSC cell invasion. The cells were grown as spheroids, as described in Material and Methods (Section 2.6). These spheroids were treated with CBG (10, 25, and 50 µM), CBD (2, 5, and 10 µM), and TMZ (100, 200, and 400 µM). The spheroids were then covered with 5 mg/mL Matrigel matrix. The invasion distance was measured after seven days for U87 cells (**A**) and after five days for U373 cells (**B**) and T98 cells (**C**) under the fluorescence microscope NIKON-Eclipse Ti at 4x magnification. The invasion area was normalized to the spheroid diameter, as determined by ImageJ software and described in the Materials and Methods (Section 2.6) [38,43]. Data are presented as mean ± S.E.M. of five to six independent experiments. Statistical analyses were performed using GraphPad Prism software, using one-way ANOVA (* *p* < 0.05, ** *p* < 0.01, and *** *p* < 0.001). Inhibition of cell invasion was observed upon CBG and TMZ treatment in all three GB cell lines and statistical significance is presented. No statistically significant decrease in cell invasion after CBD, CBD, or TMZ treatment alone was observed in the GSC line NCH421k, but the combination of CBD:CBG in the ratio of 3:1 significantly inhibited invasion by nearly 50% (**D**). Data are presented as the mean ± S.E.M. of five to six independent experiments. ** *p* < 0.01.

Enhanced GB invasion represents the important therapeutic target that could improve the treatment response. Cannabinoids have been reported to inhibit GB cell migration [24]. Thus, the inhibitory effect of the three cannabinoids was evaluated in 3D cell spheroids invasion assay. The GB lines U87, U373, and T98 were treated with increasing concentrations of CBD and CBG with varying responses below IC50 concentrations, where, above 10 µM, CBD and CBG significantly inhibited invasion by 50 to 70%, in particular in U87 cells in this experimental set-up (Figure 7A–C, Figure S3). Overall, CBD seem to be less effective inhibitor of cell invasion than CBG in all differentiated cell lines. In GSC line NCH421k, no statistically significant decrease in cell invasion after CBD treatment alone was observed. However, the CBD:CBG combination in the ratio of 3:1 significantly inhibited invasion by nearly 50% (Figure 7D).

Furthermore, the effect of CBD and CBG cell invasion in 3D cultures was compared with that of the chemotherapeutic drug TMZ, which reduced the invasion of U87 cells by approximately 50% at both 100 µM and 400 µM. However, under the same conditions, 10 µM CBG inhibited U87 cell invasion by 90%, exhibiting stronger efficacy than TMZ. In contrast, 50 µM CBG inhibited U373 cell invasion by only 50%, whilst 100 and 400 µM TMZ inhibited U373 cell invasion by 60% and 80%, respectively (Figure 7B). Both CBG and TMZ at the highest concentrations (50 µM and 400 µM, respectively) inhibited T98 cell invasion by 70% (Figure 7C). Figure S3 illustrates the spheroid assay of the dyed GB cells, where the invasive cell fraction was quantified by image analyses. In the GSC line NCH421k, CBG, CBD, and TMZ treatments alone did not decrease cell invasion. However, the CBD:CBG combination markedly and statistically significantly inhibited GSC invasion. Conversely, TMZ even increased GSC invasion (Figure 7D).

### 3.7. The Cannabinoid Receptors CB1 and CB2 are Highly but Differentially Expressed in Patient GB Cells

**Figure 8:**
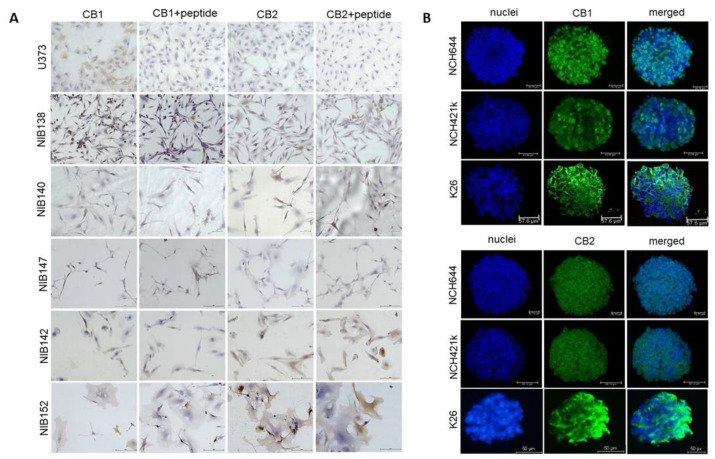
CB1 and CB2 receptors are differentially expressed in GB cells and GSCs. (**A**) Immunocytochemical staining of CB1 and CB2 receptors (brown) in GB cell lines established from patient tumour samples. Cell nuclei were counterstained with haematoxylin (blue), as described in detail in Materials and Methods (Section 2.7 and Section 2.8). Scale bars: 50 µm. (**B**) Immunofluorescence staining of CB1 and CB2 receptors in the spheroids of two established GSCs and the patient-derived GSC cell line K26. Cell nuclei were counterstained with DAPI (blue), and the receptors CB1 and CB2 were stained with Alexa Fluor 488 (green). Scale bars: 50 µm.

Based on multiple effects of CBD, CBG, and THC in differentiated GB and GB stem cells, we wanted to gain information on whether the major two cannabinoids receptors, CB1 and CB2 are expressed in the established and primary GSC cell lines, as their expression in GB has been confirmed in the literature [33,46,47,48]. We found that both CB1 and CB2 receptors were highly, but differentially, expressed in primary GB cells, indicating large patient variability in this respect (Figure 8A,B). The CB1 and CB2 receptors were also present on all GSCs at various levels (Figure 8B). This may indicate that similar mechanisms may be responsible for THC and CBD binding.

## 4. Discussion

Extracted from *Cannabis sativa* L., THC and CBD exert tumour cell-specific cytostatic effects against glioblastoma (GB) and have been mostly used in combination for the adjuvant treatment of this cancer. Numerous in vitro and in vivo studies have reported the “synergistic” effects of different combinations of these two cannabinoids. The best method to study drug combination effects would be using the Chou-Talalay method [49]. However, Deng et al. [41] pointed out that few, if any, proper kinetic tests have been performed to confirm whether the combined effects are indeed synergistic, or just additive. For example, their analyses of the combined antineoplastic activity of CBD and DNA-damaging agents suggests little improvement in their respective therapeutic indices and, in some cases, even a total loss of therapeutic efficacy, due to their antagonistic effects. Several studies have previously reported that in GB the combination of CBD and THC induces autophagy-dependent necrosis [50], apoptosis via ceramide-accumulation or by ROS activation and invasion, as summarised by Dumitri [51]; both are already in clinical testing [52]. In contrast to CBD, which has been reported to have a good safety profile [15], THC has been reported to induce dose-dependent performance impairment, as well as to increase anxiety, psychotic symptoms, heart rate, blood pressure, and to alter perception. By mixing CBD with THC, the side effects of THC were reduced, since CBD is a negative allosteric modulator of CB1, the major cannabinoid-selective receptor in the brain [53], and also antagonizes THC-mediated undesired downregulation of its anti-tumour immunity [46,47] which should be avoided [14,15]. Strategically combining the two agents in targeting GB cells by different mechanisms leading to the similar end points in tumour elimination and in general, such strategy improves the therapeutic index of certain drugs while reducing undesired side effects [24]. Using similar approaches, we focused here on testing CBG as a replacement for THC in combination with CBD.

Due to the non-intoxicating and non-psychotropic effects of CBG, an increasing number of studies are focusing on its role in different disease states [29]. As low-THC cannabis drugs are of medical interest in GB, we conducted the first study on CBG effects on GB cells in vitro. We compared the negative effect on cell viability of the purified natural CBG plant extract to that of THC and CBD in a cohort of nine patient-derived GB cell lines, three established differentiated GB cell lines, and three GSC lines. CBG consistently decreased GB cell viability in a similar concentration range (mean IC50 = 28.1 μM) to THC (mean IC50 = 27.9 μM), while CBD was significantly more cytotoxic (Table 1). This is similar in breast carcinoma, where Ligresti et al. [31] found that pure CBG was one order of magnitude less effective than CBD, but in contrast to reports by Baek et al. [28] on synthetic CBG, was found to be more cytotoxic than CBD on melanoma and oral carcinoma [54].

CBG also exerted slight cytostatic effects on GB cells, as it arrested their cell cycle in the G1 phase and decreased the percentage of cells in the S1 and G2/M phase (Figure 2). These data were complemented with significant CBG-induced GB and GSC cell apoptosis (Figure 3), which was markedly more than that of CBD, caspase-3/7-dependent. Similarly, De Petrocellis et al. [25] reported that CBG activated the proapoptotic caspase-3/7 in prostate cancer cell lines [29]. These authors [25,31] later demonstrated that CBG also inhibited prostate carcinoma growth in vitro and in vivo through TRPM8 receptor antagonism and suggested the activation of intrinsic apoptotic pathways as the possible mechanism. CBG also inhibited the carcinogenesis and growth of human colon carcinoma in mice xenographs mainly via a pro-apoptotic mechanism, which was associated with the overproduction of reactive oxygen species (ROS) [35]. Thus, the authors concluded that CBG should be considered for the prevention and treatment of colorectal cancer.

GB is a very specific type of cancer, as its metastases have rarely been found in other organs, unlike most tumours of epithelial origin that frequently metastasize to the brain [55]. This seems paradoxical, as GB cells are highly invasive and aggressively infiltrate far into the brain parenchyma, a very early event during GB growth [4,56]. Moreover, updated standard GB treatment protocols [7] may induce the expression of mesenchymal GB subtypes associated with higher invasiveness [57]. The progression and therapy resistance of cancer stem cells, which represent the root of cancer growth, have been most extensively investigated in GB. GSC resistance to conventional therapy rests on the high expression levels of active DNA damage repair and abundant xenobiotic export mechanisms. In addition, although they are potential targets for treatment, GSCs reside in therapy-protected niches, but are released as aggressive GSC/ GB cells and trigger the new growth of tumours at distant sites in the brain. However, this may also be induced after, and even by, initial therapy [57,58]. Here, we found that at an IC50 μM concentration of all three cannabinoids induced a high, approximately 30% apoptotic rate in GSCs, suggesting that this is the major reason for cytotoxicity/ cell viability inhibition. Noteworthy, this apoptosis rate is significantly higher than that induced in differentiated GB cells by CBD and CBG under the same conditions (4 to 15% at IC50 μM concentration) (Figure 3). CBG apoptotic signalling involves caspase-3/7, enhanced by the addition of CBD and TMZ. The effect of endogenous cannabinoids and their receptor signalling on human cell stemness in the ectoderm-derived nervous system has been extensively studied by Galve-Roperth et al. [48], who demonstrated that both CB1 and CB2 receptors are present in neural stem/progenitor cells and control their self-renewal, proliferation, and differentiation. CB1 and CB2 exhibit opposite patterns of expression, the former increasing and the latter decreasing during neuronal differentiation. This is consistent with the high CB1 expression in neural stem-cell-derived GSCs, which we have demonstrated by immunofluorescent in GSC spheroids (Figure 7B). CB1 and CB2 receptors were also present in all the differentiated cell lines (Figure 7A). Aguado et al. [45] first showed that GSC-like cells expressed CB1 and CB2 receptors and that cannabinoid agonists targeted the stem-like cells in a receptor-dependent manner, reducing gliomagenesis. Later, two transcription factors that regulate GSC de-differentiation and consequently lower resistance to therapy were identified. Firstly, Soroceanu et al. [59] demonstrated that CBD significantly downregulated transcription factor Id-1 gene expression, associated with glioma cell invasiveness, self-renewal, and GB stemness markers (e.g., SOX2), particularly the mesenchymal GSC subtype markers that are related to cell invasion. Secondly, Nabissi et al. [50] demonstrated that CBD treatment upregulated the expression of acute myeloid leukaemia (Aml-1) transcription factors, inducing autophagy and GSC de-differentiation, abrogating the chemoresistance of GSCs to chemotherapeutics. Along these lines, Singer et al. [60] demonstrated that, in combination with small-molecule inhibitors of antioxidant-response genes, CBD synergistically inhibited GB intracranial growth of primary GSC-derived tumours in vivo.

In contrast, a lower rate of apoptosis in differentiated GB cells was observed by all three cannabinoids, as shown here in parallel experiments. These data suggest that, apart from direct killing effects on differentiated GB cells, CBG, CBD and THC induced cell cycle arrest (Figure 2). Our data closely mirror those by Galanti [61], showing that THC holds GB cells in G0-G1 arrest, due to downregulation of Cyclin A and the E2F1 transcription factor, and upregulation of cell cycle inhibitors p16 ^INK4A^, known to drive cells to senescence and thus avoiding apoptosis. Nabissi et al. [62], also reported that THC alone and even more so THC:CBD were able to induce multiple myeloma cell accumulation in the G1 phase, in that case inducing autophagy and subsequent necrosis. Taken together, we may hypothesize that CBG, similar to cannabinoid-induced GSC apoptosis, proceeding either via cannabinoid receptor signaling or upregulated ceramide, also induces autophagy via ROS-enhanced p38 MAPK [60] and downregulated GSC stemness, as summarised by Dumitri et al. [51]. These mechanisms, in addition to differential effects on GSC vs. GB, need to be confirmed in future studies. Nevertheless, CBG can be suggested as a promising combinatorial drug candidate to target GSCs.

The differences in the effects of CBG and CBD between GSC and differentiated GB cells were further observed in our combinatorial studies of CBG and CBD. By systematically testing the cytotoxicity of all three cannabinoids in various combinations and comparing the resulting IC50 values, we have demonstrated that CBD and CBG exert an additive, but not synergistic effect on reducing GB cell viability at the optimal CBD:GBG molar ratio of 1:4. However, this ratio was suboptimal for GSC cytotoxicity where we had to increase the CBD over CBG to a three-fold molar ratio (3:1). These data clearly indicate the specificity and relevance of each cannabinoid, CBD and CBG in interactions with stem cells vs. differentiated GB cells (Figure 5A). The resistance of GSCs to lower CBD doses may be explained by an increase in mitochondrial metabolic activity due to differential binding of CBD vs. CBG to mitochondrial CB1 receptors [63,64] or by the specificity of CBD vs. CBG-induced GSC differentiation (discussed above). In any case, this effect was more pronounced than the combination of CBD:THC combinations, as shown in the representative Figure 6. Moreover, THC did not exert any additive, or even an antagonistic effect on the CBD:CBG combinations in some cases, while even the TMZ addition was also antagonistic. Our results seemingly contradict those of Thorres et al. [24] that the combined administration of THC and TMZ remarkably reduced the growth of glioma xenografts, by showing that the combined administration of THC and TMZ enhanced autophagy, playing a crucial role in their mechanism of action, but additional administration of submaximal doses of CBD induced glioma cell death through an altogether different mechanism. However, these authors have used botanical drug cannabinoids, and not purified molecules in vivo after human U87 and T98 subcutaneous injections to nude mice; therefore, the two studies are not comparable.

Finally, we explored whether combined treatment affects glioma cellular invasion, which is a highly relevant hallmark of GB progression. Successful anti-invasive therapy would not only prevent primary tumour spread that would better target GB cells, but may also prevent relapse following surgical resection. The anti-invasive effects of CBD on cancer cells have been demonstrated in numerous publications [15,65]. Here, we have demonstrated for the first time that subtoxic concentrations of CBG inhibit the invasion of GB and GSC spheroids (Figure 7). Different GB cell types (U87, T98, and U373) significantly differ in their response to the anti-invasive properties of CBG [30]. These differences may be due to the different genetic/molecular subtypes that we demonstrated to exist between U87 and U373 cells [38], and/or different levels of cannabinoid receptors in these lines [26,66]. Screening for the presence of the two major cannabinoid-selective receptors CB1 and CB2 in a plethora of GB lines and the two GSCs by semiquantitative evaluations using immunolabelling of the respective primary in vitro cell cultures, we observed that both types of the cells express both kinds of receptors, justifying patient treatment with cannabinoids. Secondly, as CB1 and CB2 differ in their expressions among patients, different responses may be expected; what explains the observed variability is their primary glioblastoma cells. Noteworthily, other non-specific ionotropic receptors may also mediate cannabinoid signaling, as reviewed by De Petrocellis et al. [26]. The mechanism of CBG inhibition of invasion may be similar to results from Soroceanu et al. [59], attributing this to the inhibition of transcription factor Id-1 expression, regulating GB invasion by CBD, and was observed in several GB cell lines, in the ex-vivo primary GB cells and in an orthotopic xenograft murine model. Alternatively, CBD treatment of GB cells may significantly downregulate metalloproteases-associated proteolytic systems. This needs to be confirmed in CBG treatment, as in our case the invasion inhibition seems to be similar as observed with CBD [30]. Additionally, upon cannabinoid binding, CB1 and CB2 may interact with GB receptors that are related to their invasion activity. For example, Nabissi et al. [62] have demonstrated that the CBD:THC combination reduced myeloma cell migration by down-regulating the expression of the important chemokine receptor CXCR4 that, along with its ligand SDF-1α (CXCL12), regulates in particular GSC homing to their niches [67] and critically affects GB progression. Coke et al. [68] reported that a CB2 agonist specifically reduced CXCR4-mediated migration. Clinically, CXCR4 expression in tumours is used to predict cancer aggressiveness [43], and the CXCR4 blocking ligand plerixafor successfully inhibited the progression of acute myeloid leukaemia (Aml I-a) in a clinical setting [69].

Interestingly, we found that the chemotherapeutic drug TMZ at 400 µM reduced the invasion of U87 cells by approximately 50%, U373 cell invasion by 70 %, and T98 cell invasion by 80%. Conversely, in the GSC line, NCH421k TMZ treatment highly increased GSC invasion (Figure 7D). These preliminary, though consistent, data are difficult to interpret. TMZ is known to methylate nuclear DNA guanine residues at several positions via complex mechanisms, resulting in cytostatic G2/M cell cycle arrest and autophagy-induced apoptosis, depending on the cell context [70], which may not be detected by MTT assays alone. Of note, TMZ also potentially alkylates mitochondrial DNA and RNA, as well as proteins and lipids carrying nucleophilic groups, acting at post-transcriptional/post-translational levels. Although TMZ is a standard chemotherapeutic, used in the treatment of GB patients at much lower concentrations (14.95–34.54 μM) [70], highly different concentrations (100 and 4000 μM) were required to be effective in the in vitro models. Altogether there is still much debate surrounding TMZ’s mechanisms and its efficacy in clinical use [9], taken together, our data corroborate the statement by Deng et al. [41] on the dichotomy of the antiproliferative and apoptotic effects of TMZ in GB.

## 5. Conclusions

This is the first report to demonstrate that the non-intoxicating cannabinoid CBG alone and in combination with CBD efficiently targets two key elements that otherwise prevent the successful treatment of GB patients with current therapeutics: Firstly, to overcome GSC resistance to cytotoxic agents and to induce apoptosis, and secondly, to inhibit GB cell invasion. Our findings on the effects of cannabinoids on GSCs are consistent with previous observations that demonstrate that cannabinoid receptor agonists promote glial differentiation by altering the expression of genes involved in the regulation of stemness. As such, differentiated GB cells were also exposed to the cytotoxic, proapoptotic, and anti-invasive effects of CBG. We have demonstrated that the combination of CBG and CBD, each at sub-cytotoxic concentrations, results in additive effects on reduced cell viability and induced apoptosis, which are sufficient to replace the application of THC. Formulations containing THC are thus not needed and could be avoided due to the psychoactive activity of THC that is particularly harmful to GB patients with neurological distortions associated with tumour progression. Furthermore, besides exerting clear antitumour effects, CBG and CBD also exert known palliative off-target effects, such as analgesia, increased appetite, and the prevention of chemotherapy-induced cachexia and nausea.

## Figures and Tables

**Table 1:** The inhibitory effects of CBG, CBD, and THC on glioblastoma (GB) and glioblastoma stem cell (GSC) viability, calculated as IC50 (μM) *.

Cell Lines (GB) **	CBG [µM]	CBD [µM]	THC [µM]
U87	24.2	17.4	25.5
U373	31.1	29.7	34.9
NIB138	22.3	18.3	19.5
NIB140	32.0	26.7	29.9
NIB142	27.4	25.9	34.4
NIB160	26.3	15.0	21.1
NIB167	25.6	12.6	23.7
NIB180	30.8	20.1	29.6
NIB182	31.6	28.1	30.2
NIB185	29.8	28.8	30.0
Mean ± S.E.M.	28.1 ± 1.1	22.2 ± 2.1	27.9 ± 1.8
**Stem Cell Lines (GSCs)**	**CBG [µM]**	**CBD [µM]**	**THC [µM]**
NCH644	58.3	15.9	22.3
NCH421k	34.0	27.9	28.7
K26	84.8	14.6	17.4
Mean ± S.E.M.	59.0 ± 14.7	19.5 ± 4.2	22.8 ± 3.3

* IC50 values (expressed in μM) for CBG-, CBD-, and THC-treated GB cells and GSCs were calculated from the half maximal inhibitory effects on GB and GSC viability using GraphPad Prism software, as described by Deng et al. [41] and in detail in the Methods section. Each value represents the mean of three independent biological assays (individual S.E.M. are shown in Figure 1). ** Besides commercially available cell lines, we have used patient-derived cell lines stablished at the **N**ational **I**nstitute of **B**iology (**NIB**) laboratory, labeled as NIB lines, systematically collected from a tumour bank termed “Gliobank” that is an international (Slovenia-Italy) glioma tissue bank (see Section 2.3).

## Data Availability

Not applicable.

## Supplementary Materials

The following are available online at https://www.mdpi.com/2073-4409/10/2/340/s1, Figure S1: Biosynthesis and metabolism of cannabigerol (CBG), Figure S2: Cell death determination by flow cytometry using Annexin V-FITC and propidium iodide staining, Table S1: High-performance liquid chromatography purity results for the CBG, CBD, and THC solutions used in this study.
