# Supplementary material for: Cannabigerol Is a Potential Therapeutic Agent in a Novel Combined Therapy for Glioblastoma
Source: Cells. 2021 Feb 5;10(2):340. doi: 10.3390/cells10020340 (PMC7914500; doi:10.3390/cells10020340)
Supplement: Supplementary file 1 [file cells-10-00340-s001.pdf]

cells

MDPI

Supplemental material

Cannabigerol is a potential-therapeutic agent in a novel combined therapy for glioblastoma

Tamara T. Lah, Metka Novak, Milagros A. Pena Almidon, Oliviero Marinelli, Barbara Žvar Baškovič, Bernarda Majc, Mateja Mlinar, Roman Bošnjak, Barbara Breznik, Roby Zomer, and Massimo Nabissi

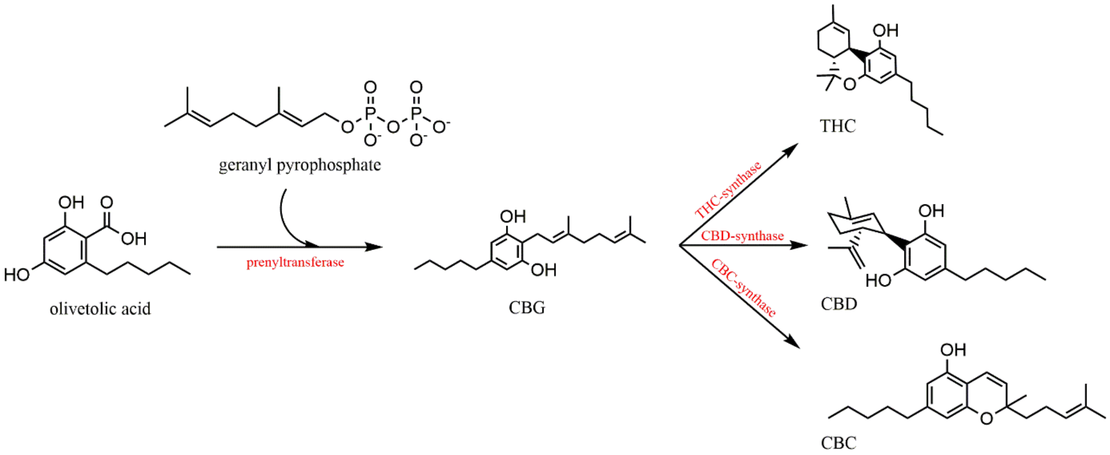

Figure S1. Biosynthesis and metabolism of cannabigerol (CBG). The enzyme prenyltransferase catalyses the conversion of olivetolic acid into CBG in the cannabis plant. CBG is the intermediate biosynthetic precursor of delta-9-tetrahydrocannabinol (THC)-acid and cannabidiol (CBD)-acid and is converted to THC, CBD, and cannabichromene (CBC), which is then converted into THC, CBD, or CBG by specific synthases (adapted from [30]).

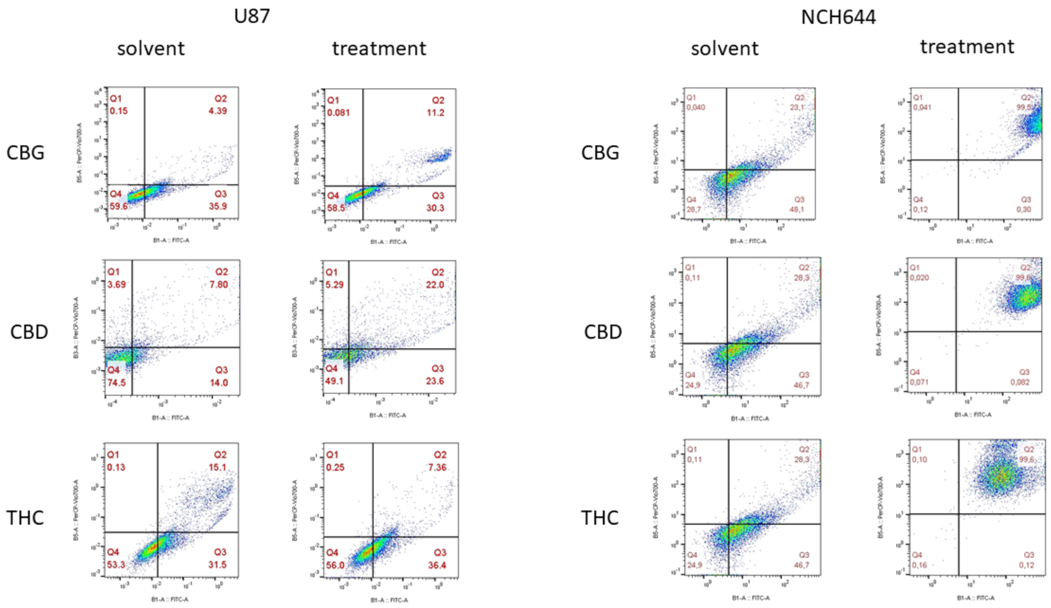

Figure S2. Cell death determination by flow cytometry using Annexin V-FITC and propidium iodide staining. Cells (U87 and NCH44) were treated with IC_{50} concentrations of compounds CBG, CBD, and THC for 48 h. Cells were labelled with Annexin V-FITC and propidium iodide and analysed by flow cytometry. The dot blots represent the results from three biological repeats.

Table S1: High-performance liquid chromatography purity results for the CBG, CBD, and THC solutions used in this study.

Cannabinoids in CBG solution	Concentration (mg/ml)	Cannabinoids in CBD solution	Concentration (mg/ml)	Cannabinoids in CBD solution	Concentration (mg/ml)
CBDVA	BDL*	CBDVA	BDL*	CBDVA	BDL*
CBDV	BDL*	CBDV	BDL*	CBDV	BDL*
CBDA	BDL*	CBDA	BDL*	CBDA	BDL*
CBGA	BDL*	CBGA	BDL*	CBGA	BDL*
CBD	BDL*	CBD	1484	CBD	BDL*
CBG	5810	CBG	BDL*	CBG	BDL*
THCV	BDL*	THCV	BDL*	THCV	BDL*
CBN	BDL*	CBN	BDL*	CBN	BDL*
delta-9-THC	BDL*	delta-9-THC	BDL*	delta-9-THC	1524
delta-8-THC	BDL*	delta-8-THC	BDL*	delta-8-THC	BDL*
CBL	BDL*	CBL	BDL*	CBL	BDL*
CBC	BDL*	CBC	BDL*	CBC	10
THCA	BDL*	THCA	BDL*	THCA	BDL*

BDL*: below detection limit, CBDVA: cannabidivarinic acid, CBDV: cannabidivarin, CBDA: cannabidiolic acid, CBGA: cannabigerolic acid, CBD: cannabidiol, CBG: cannabigerol, THCV: tetrahydrocannabivarin, CBN: cannabinol, delta-9-THC: delta-9-tetrahydrocannabinol, delta-8-THC: delta-8-tetrahydrocannabinol, CBL: cannabicyclol, CBC: cannabichromene, THCA: tetrahydrocannabinolic acid.

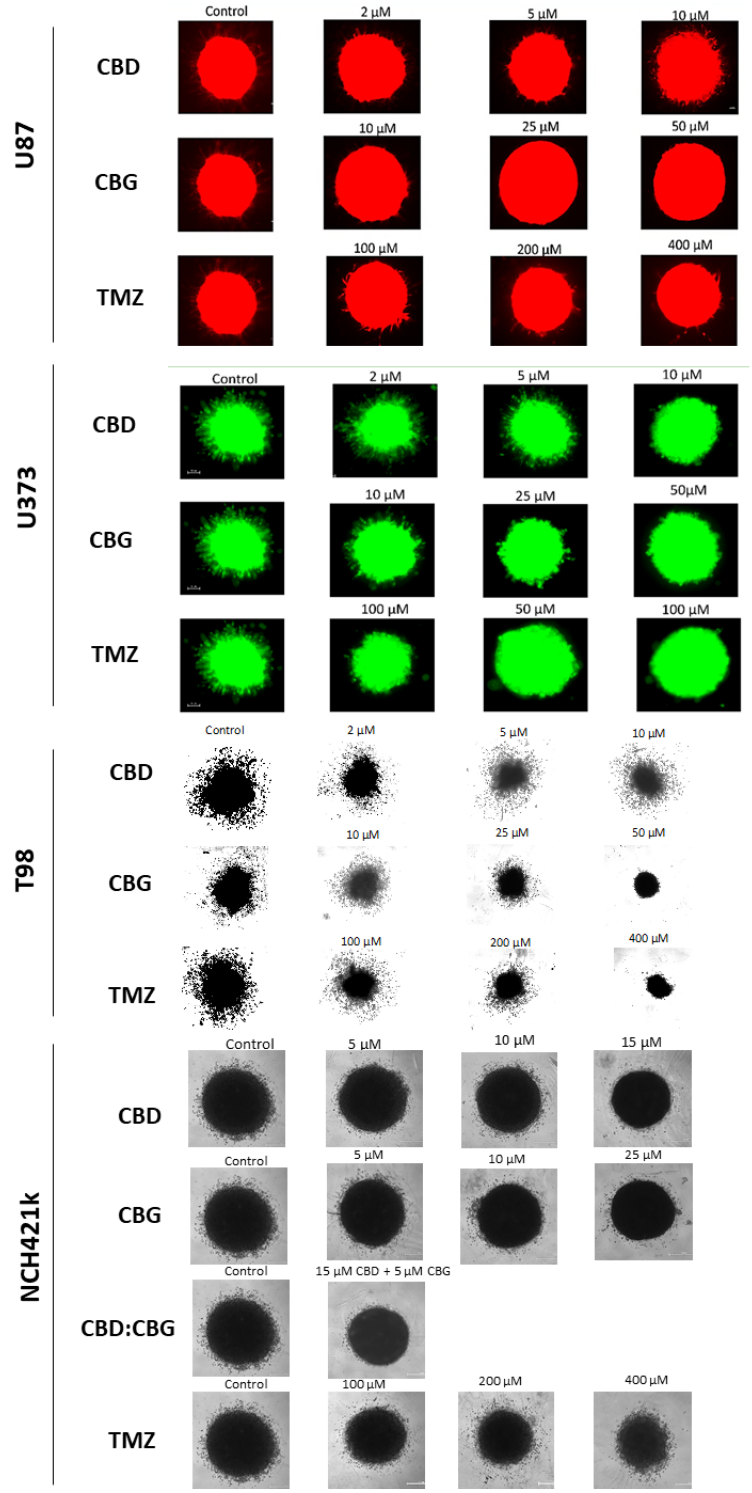

Figure S3: The effect of CBD, CBG and TMZ on U87, U373 and T98 GB and NCH421k GSC cell invasion. Figure S3 is illustrating the representative spheroid assay of the dyed GB cells, where the invasive cell fraction was quantified by image analyses. Quantified analysis of the effects of cannabinoids CBD and CBG on GB and GSC invasion, are presented in graphs Figure 7.
